# The GC Content as a Main Factor Shaping the Amino Acid Usage During Bacterial Evolution Process

**DOI:** 10.3389/fmicb.2018.02948

**Published:** 2018-12-07

**Authors:** Meng-Ze Du, Changjiang Zhang, Huan Wang, Shuo Liu, Wen Wei, Feng-Biao Guo

**Affiliations:** ^1^School of Life Science and Technology, University of Electronic Science and Technology of China, Chengdu, China; ^2^School of Life Sciences, Chongqing University, Chongqing, China; ^3^Centre for Informational Biology, University of Electronic Science and Technology of China, Chengdu, China

**Keywords:** amino acid composition, amino acid cost, bacteria, evolutionary rate, last universal common ancestor, GC content, metabolic efficiency

## Abstract

Understanding how proteins evolve is important, and the order of amino acids being recruited into the genetic codons was found to be an important factor shaping the amino acid composition of proteins. The latest work about the last universal common ancestor (LUCA) makes it possible to determine the potential factors shaping amino acid compositions during evolution. Those LUCA genes/proteins from *Methanococcus maripaludis* S2, which is one of the possible LUCA, were investigated. The evolutionary rates of these genes positively correlate with GC contents with *P*-value significantly lower than 0.05 for 94% homologous genes. Linear regression results showed that compositions of amino acids coded by GC-rich codons positively contribute to the evolutionary rates, while these amino acids tend to be gained in GC-rich organisms according to our results. The first principal component correlates with the GC content very well. The ratios of amino acids of the LUCA proteins coded by GC rich codons positively correlate with the GC content of different bacteria genomes, while the ratios of amino acids coded by AT rich codons negatively correlate with the increase of GC content of genomes. Next, we found that the recruitment order does correlate with the amino acid compositions, but gain and loss in codons showed newly recruited amino acids are not significantly increased along with the evolution. Thus, we conclude that GC content is a primary factor shaping amino acid compositions. GC content shapes amino acid composition to trade off the cost of amino acids with bases, which could be caused by the energy efficiency.

## Introduction

Amino acid composition reflects the usage of twenty standard amino acids in proteins. Understanding the changes of amino acid composition among homologous proteins is key to the investigation of protein functioning, as the proteins can acquire new functions through amino acid substitutions (Misawa et al., 2008). The amino acid compositions vary among proteins, even among those homologous proteins. The amino acid composition was reported to be correlated with the protein structure classes (Bahar et al., 1997; Horner et al., 2008; Du et al., 2014), the metabolic efficiency (Akashi and Gojobori, 2002; Kaleta et al., 2013), and the translation efficiency (Du et al., 2017). Sueoka (1961, 1962) firstly reported that there is a correlation between GC contents and amino acid composition of proteins, and then the nucleotide bias causes the biased amino acid usage in bacterial and viral genomes was broadly reported (Rooney, 2003; Bohlin et al., 2013; Goswami et al., 2015). Cost-minimization could also shape the amino acid composition (Seligmann, 2003; Raiford et al., 2008; Bivort et al., 2009). Another factor, which influences the amino acid gain and loss in protein evolution and thus causes the biased amino acid usage is the order of amino acids being recruited into the genetic codes (Jordan et al., 2005; Hurst et al., 2006; Mcdonald, 2006; Liu et al., 2015). However, we still do not know how the feature of amino acids contributes to shape their biased compositions in proteins.

Life emerged and has been evolving, and the imprint of evolution is recorded by the genomes (Martin et al., 2016). If the amino acid composition of early life is known, it is possible to infer the factors that cause the biased amino acid usage of proteins during the evolution process. Brooks and Fresco (2002) analyzed the amino acid frequencies in extant proteomes and found that the frequencies of several amino acids increased since the divergence of the last universal common ancestor (LUCA). The LUCA, which could be inferred by comparing the genomes of its descendants, is the most recent ancestor from which all currently living species have evolved. Weiss et al. (2016) traced the LUCA by phylogenetic criteria and identified a set of genes from 355 families, which implies a very specific lifestyle. This work places *clostridia* and *methanogens* as the earliest-diverging organisms, which provides us with a very intriguing insight into the LUCA (Mcinerney, 2016). The hydrogenotrophic methanogenic archaeon *Methanococcus maripaludis* S2 (MmarS2) is a well-studied organism which is anaerobic, H_2_-dependent and uses the Wood-Ljungdahl pathway (Goyal et al., 2014). Thus, it is possible to choose this organism as one representative of LUCA to investigate the variation of amino acid frequencies.

Because most essential genes are ancient and more evolutionary conserved (Jordan et al., 2002; Chen et al., 2010), we used essential genes as a representative set of ancient genes and observed the amino acid composition of corresponding proteins homologous to those proteins of MmarS2. Firstly, it is shown that in these protein coding genes GC contents have more significant effects on the amino acid deviation than the amino acid recruitment order with LUCA protein and non-LUCA proteins. Secondly, the gain and loss of amino acids for these homologous proteins do not accord well with the amino acid recruitment orders. Thus, the GC variations have more effects on the amino acid usage bias than the recruitment order of amino acids. The GC content influence the amino acid composition maybe caused by the energy efficiency.

## Results

### Homologous Proteins to LUCA and Non-LUCA Proteins of MmarS2

Weiss et al. (2016) determined that the earliest-diverging organisms belong to *clostridia* or *methanogen*, and they supplied a list of genes which were putative LUCA genes. *M. maripaludis* S2 (MmarS2) is the only methanogen organism whose essential genes were determined experimentally (Luo et al., 2014). The LUCA gene list contains 195 MmarS2 Clusters of Orthologous Groups of proteins (COGs). Only three sequenced bacterial genomes have more consistent COGs to that of LUCA genes than MmarS2 (Supplementary Table S1). *Methanosarcina acetivorans* C2A uid57879 has 211 LUCA COGs, *Streptosporangium roseum* DSM 43021 uid42521 has 201 ones and *Methanosarcina barkeri Fusaro* uid57715 has 198 ones. Additionally, the living environment of MmarS2 is similar to the predicted living condition of LUCA. Thus, MmarS2 could be a good representative of LUCA.

**FIGURE 1:**
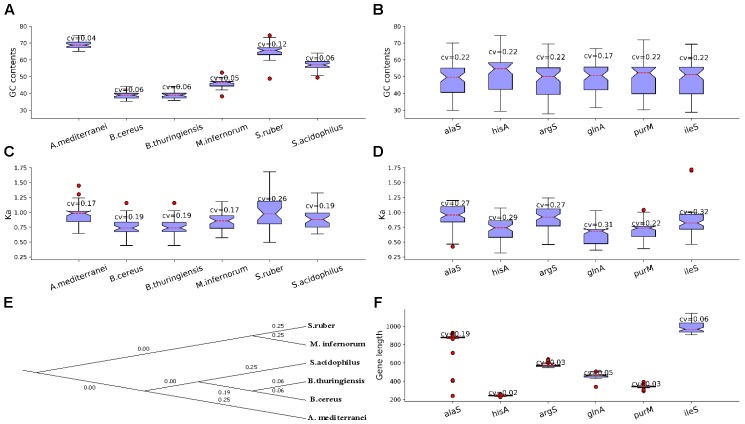
Genes have biased GC contents and evolutionary rates in and among genomes. **(A)** The GC content of genes homologous to LUCA genes. The CV is short for Coefficient of Variation, which equals to standard deviation(x)/mean(x), while x is the list of GC contents of those genes in the LUCA list. Although homologous genes originate from the same gene and have identical GC contents in the ancestor, these genes tend to evolve to have preferred GC contents for the genomes. **(B)** Genes evolve to have variable GC content among genomes. By observing the GC content for evolved genes in genomes and among genomes, **(A,B)** show that GC content may play an important role for genome evolution. **(C)** The *Ka* of gene stes homologous to genes in the same genomes tend to be less discrete than that of the gene sets which contain homologous genes from different genomes **(D)**, which means genes in one genome have similar evolutionary rates, while the genes among genomes evolve biasedly. **(E)** Shows the evolutionary relationship between the six presented organisms with *K* = 6, while using the MmarS2 as outgroup. **(F)** The six genes have different gene lengths. We chose these genes randomly, thus the evolutionary results may be not correlated with the gene length.

Next, we listed some LUCA genes/proteins and non-LUCA genes/proteins for further analysis. There are 520 essential genes determined under rich medium for MmarS2, and 85 ones of them are included in the list of 355 LUCA genes which belong to 21 types of functional categories (Weiss et al., 2016). The 85 essential genes of MmarS2 which will be seen as the representative of LUCA genes belong to 17 different functional categories. The four LUCA functional categories not included in the further analysis are translation (2 COGs), energy metabolism, protein modification, and cell wall related (Supplementary Table S2). In contrast to non-LUCA genes, the 85 essential genes which could be seen as the representative of LUCA genes supposed to be more conserved and ancient (Yin et al., 2016). Then, we determined their homologous genes from other organisms. We chose bacterial and archaea genomes with at least 10 genes having the gene name in the above LUCA gene list for further analysis. Consequently, 527 genomes among 2000 sequenced bacteria and archaea that met this criterion were included into the analyses (Supplementary Table S3). The same gene as those in the representative LUCA gene list tends to have variable GC content preferred by corresponding organisms, and we showed this tendency in six model genomes/genes (Figures 1A,B). The six genomes are *Amycolatopsis mediterranei* U32 uid50565 (*A. mediterranei*), *Bacillus cereus* E33L uid58103 (*B. cereus*), *Bacillus thuringiensis* serovar konkukian 97 27 uid58089 (*B. thuringiensis*), *Methylacidiphilum infernorum* V4 uid59161 (*M. infernorum*), *Salinibacter ruber* M8 uid47323 (*S. ruber*) and *Sulfobacillus acidophilus* TPY uid68841 (*S. acidophilus*). They have more homologous proteins with the LUCA protein set than other genomes. Genes of the six model organisms have different GC contents, while all the GC contents/evolutionary rate (*Ka*) of protein coding genes in these genomes have small coefficient variations (0.04∼0.12; 0.17∼0.26) (Figures 1A,C). The six genes exist in more than 58 genomes. *IleS* is isoleucyl-tRNA synthetase, *purM* is phosphoribosylaminoimidazole (AIR) synthetase, *glnA* is Glutamine synthetase, *argS* is arginyl-tRNA synthetase, *hisA* is uncharacterized protein related to proFAR isomerase and *alaS* is alanyl-tRNA synthetase. The GC contents for the six protein-coding genes alaS, ileS, argS, hisA, glnA and purM from MmarS2 are 35.91, 34.65, 34.69, 33.88, 39.52, and 33.56%. The GC contents/evolutionary rates of homologous genes have high coefficient variation CV (0.22; 0.22∼0.32), which means the genes with same name evolve to have a very different GC content close to the average content of the genome in which it exists (Figures 1B,D). The evolutionary tree of the six model organisms shows that they have different evolutionary distances to the MmarS2 (Figure 1E). All six LUCA genes with different gene lengths are randomly chosen (Figure 1F).

However, the LUCA and non-LUCA may suffer the same evolutionary stress. Thus, we also listed the homologous proteins of 90 non-LUCA proteins of MmarS2, which are not in the list of the predicted LUCA genes by Weiss et al. (2016), were also determined (Supplementary Table S4). These non-LUCA has the similar CV values of GC content and *Ka*. Thus, these homologous LUCA and non-LUCA proteins may both be helpful to inspect how the GC content together with other factors influences the evolutionary process of genomes.

### Proteins Evolve Among Genomes Under Strong Effects of GC Content

From the above results, we observed that genes in the same genomes tended to evolve to have similar GC contents. The homologous genes from different genomes have various GC content. Thus, the effect of GC content on protein evolution could be important. Previous researches reported that local GC content largely influences the recombination rates (Fullerton et al., 2001), and the GC content is mainly determined by the interactions among gene structure, recombination patterns, and GC-biased gene conversion (Glémin et al., 2014). Furthermore, Sueoka reported that amino acid compositions correlate with GC contents in few bacterial genomes before the genetic codons being elucidated (Sueoka, 1961, 1962). It was also reported that amino acid usage and GC content also shape each other (Lightfield et al., 2011; Khrustalev and Barkovsky, 2012). Here we tried to investigate the effects of GC content on amino acid composition during evolutionary process through LUCA proteins and non-LUCA proteins in bacteria.

**FIGURE 2:**
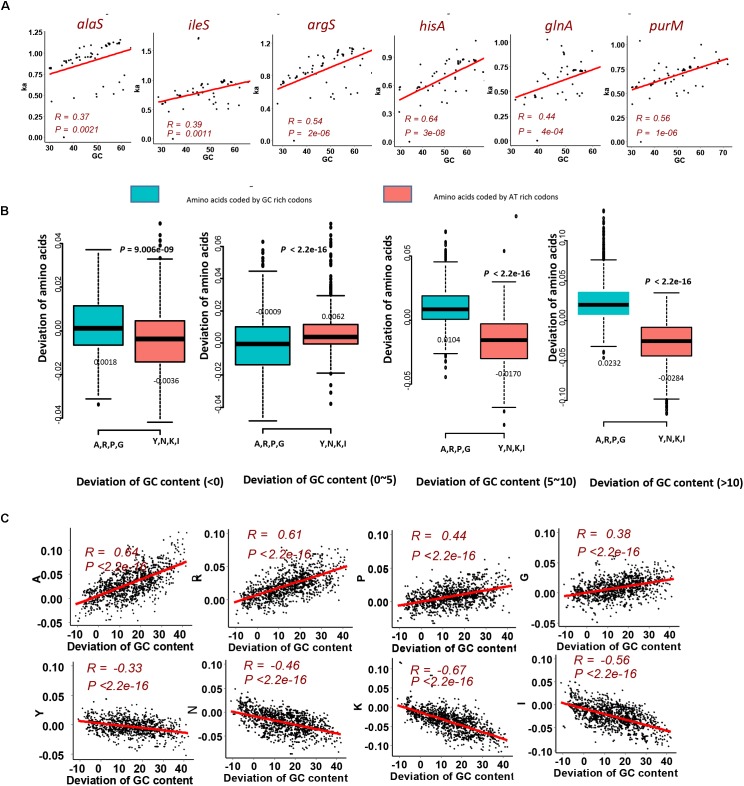
GC content significantly contributes to the LUCA protein evolution. **(A)** Evolutionary rate (*Ka*) for six genes homologous to the LUCA genes from multi genomes positively correlates with corresponding GC content. Higher GC content happens with higher *Ka* value. The red points are genes from MmarS2. **(B)** The gain and loss tendency of amino acids encoded by the GC rich/AT rich codons under different GC deviations. We used the GC content of one gene to minus the GC content of its homologous gene in MmarS2, and then we acquire the deviation of GC content. The deviation of amino acids is the amino acid frequencies of one gene minus the amino acid frequencies of its homologous gene in MmarS2. More details can be found in the part of Section “Materials and Methods.” **(C)** The correlation between the deviation of GC content and the deviation of amino acid composition. Amino acids A, R, P, and G are encoded by GC rich codons, while amino acids Y, N, K, and I are encoded by AT rich ones.

To investigate that the GC content has effects on amino acid compositions of homologous genes, we firstly calculated the change of frequencies for the twenty standard amino acids in genes mentioned above, which are essential genes of MmarS2 and are present in more than sixty bacterial strains. For 47 ones among 50 genes who have more than seven homologous genes, their GC contents positively correlate with the corresponding *Ka* values (Figure 2A, *P* < 0.005; Table 1). Only three genes rfaG, nifH, and selD do not have significant correlation between evolutionary rates and GC contents. GC contents were growing along with *Ka* values under different linear determination coefficients.

**Table 1:** The correlation between evolutionary rates and GC content.

Gene name	*R*	*P*	Gene name	*R*	*P*
fni	0.2321	2.4372E–02	purM	0.6882	3.3737E–68
thiH	0.8805	1.7266E–03	pheT	0.4847	1.0511E–12
ssuD	0.4681	2.1050E–02	rpsK	0.6360	5.0975E–57
hemA	0.6683	6.7149E–51	rpsI	0.7290	1.1689E–68
cysG	0.4130	6.2786E–02	ylqF	0.9256	2.7882E–03
**rfaG**	**0.0309**	**8.4614E–01**	uppS1	0.6528	4.4967E–03
hypF	0.8290	9.9017E–43	fusA	0.4858	2.9113E–28
**nifH**	**0.0876**	**4.6742E–01**	rpsJ	0.7263	4.3783E–84
leuA	0.4844	5.4731E–24	rplN	0.2635	1.7446E–09
glyS	0.6225	5.2589E–14	rplE	0.5115	3.7355E–35
gldA	0.6756	2.0399E–13	rpsH	0.2731	3.5679E–10
nrdD	0.2517	1.3107E–04	rpsE	0.7636	1.4015E–98
rplA	0.6025	1.2137E–49	Dcd	0.5760	2.8708E–28
alaS	0.6007	5.1704E–52	ileS	0.6564	4.1183E–68
prsA	0.2571	1.5232E–05	codA	0.9621	8.6068E–06
gcp	0.4890	3.1475E–06	pyrE	0.5863	1.1862E–44
tpiA	0.2963	3.5505E–03	pheS	0.6598	1.3323E–20
proS	0.3768	3.2182E–04	hypB	0.6436	2.5365E–20
spoVB	0.9663	1.2079E–18	pheA	0.5966	2.1879E–38
gatB	0.5897	6.8656E–38	**selD**	**0.4904**	**5.3793E–02**
lysC	0.4637	4.4213E–17	rplB	0.7030	2.2372E–77
argS	0.6057	3.5711E–54	Ffh	0.3552	1.9826E–23
sun	0.8789	4.3642E–28	speE	0.2516	1.3146E–04
mch	0.6761	4.0335E–03	trkA	0.4325	2.3957E–03
glnA	0.3781	2.8890E–18	hisA	0.7805	9.9555E–80

*The items which have bad correlation between GC content and Ka are marked in red. These genes rfaG, nifH and selD have homologous genes in 42, 71 and 16 genomes.*

We further observed that sizes of the genomes containing these homologous genes also positively correlate with the GC contents of these homologous genes (Supplementary Figure S1). For different genes, the amino acid compositions significantly influencing the evolutionary rates are different. Considering that huge genomes have more proteins to be translated. Amino acids have different energy and material cost for synthesis, which means some amino acids are much more expensive than others. Thus, huge genomes incline to employ cheaper amino acids, while cheap amino acids tend to be GC rich and genes with higher GC content tend to be highly expressed (Chen et al., 2016). Amino acid composition reflects the action of natural selection to enhance metabolic efficiency, and cheaper amino acids tend to be encoded by codons with high GC content. Consequently, we observe the positive linear relationship between GC content and *Ka* values, which may be caused by maximizing the metabolic efficiency.

Although some of genes from MmarS2 (ileS vs. argS; hisA vs. purM) show similar GC contents, the amino acids whose compositions contribute to the evolutionary rates vary a lot according to the Ridge regression results (Table 2). Linear regression models were constructed between evolutionary rates (*Ka*) and amino acid compositions of LUCA as well as non-LUCA proteins using principal analysis regression (Supplementary Table S4) to solve the multilinear problem. Firstly, six principal components are extracted from 20 amino acid composition values and then linear regression was performed between the predicted values of the six principal components and the *Ka* values. The linear models show that amino acid compositions significantly contribute to the evolutionary rates (>29%, *P* < 0.0005). All the first principal component for these homologous genes has a very high correlation with their GC contents (The mean of average |*R*| is 0.85, *P* < 0.0005). Amino acids encoded by GC-rich codons frequently existing in the first principal component show that the first principal component represents the GC-content and thus GC contents decide the evolutionary rate as the main factor.

**Table 2:** The linear models between evolutionary rates and amino acid compositions.

Gene name	Linear regression models (Ridge regression)		Linear regression models (Principal component regression)	
	*R*^2^	Variables significantly contribute to evolutionary rates (positive; negative)	*R*^2^	Amino acid significantly contribute to the first Principal component (positive; negative)
alaS	0.93	F,H,T; Y,M,W,E,C,D,I,P	0.7	A,D,G,H,S,R; C,E,I,K,N,Y
ileS	0.93	A,G,Q; D,C,V,W	0.84	A,D,G,M,P,R,W; E,F,I,K,L,N,S,Y
argS	0.77	Q; S,E,I	0.69	A,D,G,H,M,Q,P,R,T; C,E,F,I,K,N,S
hisA	0.88	Y,L,R,A; D,M,E	0.77	A,D,G,H,L,Q,P,R,T; E,F,I,K,M,N,S
glnA	0.64	S,A,C,Q; D	0.46	A,G,H,P,S,T; C,E,F,K,L,N
purM	0.72	P,N; H,T,I	0.55	A,D,G,H,L,Q,P,R,T,W,V; E,F,I,K,M,N,Y

*All multiple linear models in this table have P-values less than 1.539E-06. Amino acids coded by GC-rich codons are A, P, R, and G, while amino acids coded by AT-rich codons are F, I, N, K, M, and Y.*

Next, to detect the detailed amino acid gain and loss during evolution according to GC content, we investigated the composition variation of GC rich amino acids and AT rich amino acids for homologous proteins. Comparing with proteins of MmarS2, amino acid contents may increase or decrease in corresponding homologous proteins. According to the GC content deviation degree we classify the homologous genes from different genomes into four groups. Genes with increased GC content, especially those with GC content being 10% higher than that of homologous genes belonging to MmarS2 (deviations of GC contents: >10), have higher ratios of GC rich amino acids and lower ratios of AT rich amino acids (Figure 2B). The deviation of amino acid composition for GC rich amino acids positively correlates with the deviation of GC content, while the deviation of amino acid composition for AT rich amino acids negatively correlates with the deviation of GC content (Figure 2C). Finally, it can be deduced that genomes with higher GC content may have higher ratios of GC rich amino acids.

In conclusion, the LUCA proteins evolve under strong effects of GC content, which probably is selected by metabolic efficiency of amino acids. GC rich amino acids tend to increase along with the increase of GC content of protein coding genes.

### Effect of the Recruitment Order of Amino Acids on the Evolution Tendency of Amino Acid Composition

It is proved that GC content is one strong factor for promoting protein evolution and shaping the amino acid composition. However, the recruitment order of amino acids was considered as a main component deciding the mutation direction of amino acids (Jordan et al., 2005). The GC features for each amino acid were determined based on corresponding genetic codons (Osawa et al., 1992), and feature values for the recruitment order, cost, and molecular weight were also acquired from previous researches (Akashi and Gojobori, 2002; Jordan et al., 2005). According to the feature values to each amino acid (Supplementary Table S6), correlation analyses were performed and GC contents negatively correlate with the recruitment order of amino acids (*R* = −0.60, *P* = 0.005), the recruitment order positively correlate with the cost (*R* = 0.78, *P* = 5.483E–05), and the cost and molecular weight correlate well (*R* = 0.7942, *P* = 4.899E–05). The correlation analysis between these features show that GC content could have contradictory effects on evolutionary rates than cost, molecular weight, and recruitment order. The energy cost for synthesizing amino acids (Akashi and Gojobori, 2002) and the molecular weight of amino acids also are important factors influencing the evolutionary rates. However, their correlation with the gain and loss of amino acid is very weak (not significant). Then, we investigated the effects of amino acid recruitment order on the evolutionary rates. Sixty-five genomes were used in the following analysis. Firstly, it is investigated that whether the ancient amino acids have higher frequency than the newly recruited amino acids in all these researched genomes. The contents for ancient amino acids and newly recruited ones in proteins were compared, and the result showed that the ancient ones do have higher ratios than that of the newly recruited ones (Supplementary Figure S2, students’ *t*-test: *P* < 2.2E–16).

**FIGURE 3:**
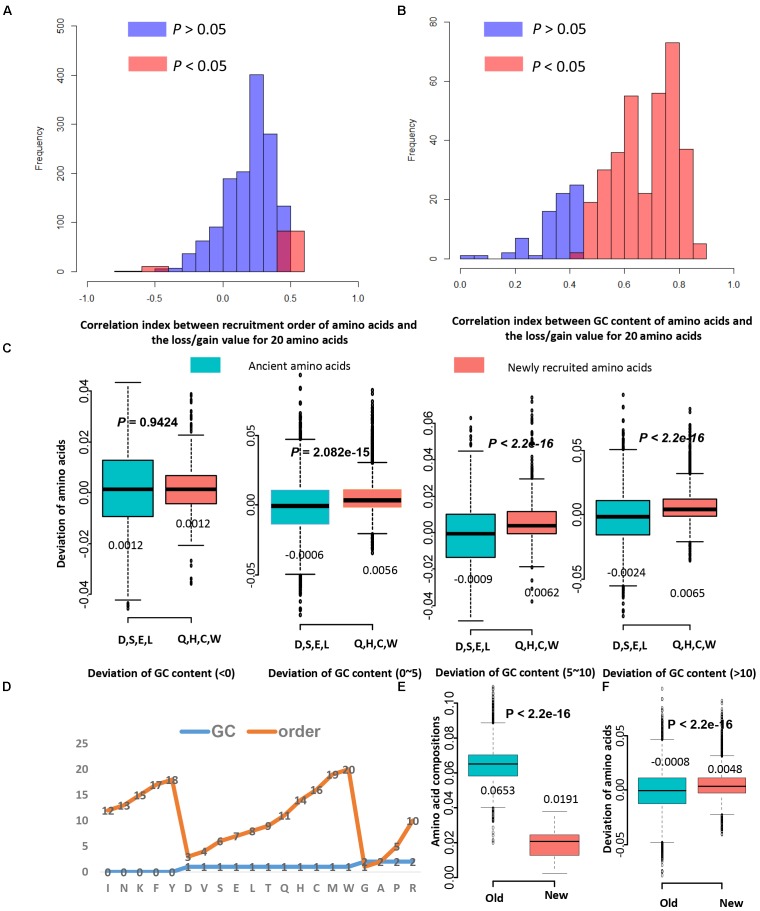
The recruitment order of amino acids contributes less to the LUCA protein evolution than GC content. **(A)** The histograms for correlation indexes between recruitment order of amino acids and the loss/gain value for 20 amino acids. Only in 19.33% proteins (1501 records from 65 genomes) the deviation of amino acids correlates with the amino acid recruitment order. **(B)** The histograms for correlation indexes between GC content of amino acids and the loss/gain value for 20 amino acids. In 62.73% proteins the GC content significantly correlates with the deviation of amino acids. **(C)** The boxplots of values of deviation of amino acids for ancient amino acids and newly recruited amino acids. Four ancient amino acids D, S, E, L (Asp, Ser, Glu, Leu) tend to be lost during evolution in proteins homologous to LUCA proteins, while four newly recruited amino acids Q, H, C, W (Gln, His, Cys, Trp) tend be gained. **(D)** The order and GC content features for 20 standard amino acids. The GC-rich amino acids are with values of 2, and the AT-rich amino acids are with values of 0. The early recruited amino acids are from 1 to 10, and the newly recruited amino acids are from 11 to 20. **(E)** The amino acid compositions for old amino acids (D, V, S, E, L, T) are significantly higher than that of new amino acids (Q, H, C, W) for 2774 bacteria and archaea genomes. **(F)** The deviation of amino acids for old amino acids (D, V, S, E, L, T) are significantly lower than that of new amino acids (Q, H, C, W) for 1501 proteins. These proteins were used in panels **(A,B)**. Although new amino acids have lower amino acid levels, they may have higher deviations. Thus, the low levels of new amino acid compositions may not cause the lower chance of amino acid replacement occurrence.

**FIGURE 4:**
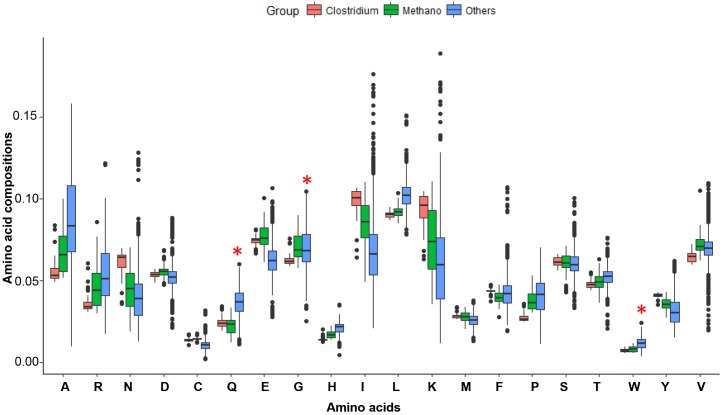
The amino acid compositions for possible LUCA organisms and all other microorganisms. Clostridium (48 genomes) and Methano (51 genomes) are possible LUCA organisms, and Others (2675 genomes) are some bacteria and archaea genomes. Amino acids Q, H, C, M, and W are new amino acids and encoded by codons at average GC contents. The Methano group has less Q, H, W amino acids than others. Amino acids C and M of Methano and Clostridium are a little higher may because they have more sulfur or living in a circumstance without higher sulfur. The Methano here is short for Methanoproducents.

Next, it is investigated whether the recruitment order influence the gain and loss of amino acids during evolution. The correlation analysis result showed that only in 19.33% proteins (1501 records from 65 genomes) the deviation of amino acids correlates with the amino acid recruitment order (Figure 3A). For comparison, it was shown that in 62.73% proteins the GC content significantly correlate with the deviation of amino acids (Figure 3B). The GC content of amino acids have more effects on amino acid composition than the recruitment order of amino acids. To further verify this, the gain and loss conditions for those earliest and latest four amino acids in homologous proteins with various GC variation were compared (Supplementary Table S5). Four eldest amino acids (Asp, Ser, Glu, and Leu) tend to be lost more often than being gained, while four newest amino acids (Gln, His, Cys, and Trp) tend to be gained more during evolution process (Figure 3C). When the change range of GC content is 0 to 5 percent, old amino acids incline to be lost, while new amino acids incline to be gained. When the change range of GC content is higher than five percent, compositions of these amino acids tend to be largely influenced by the GC content. The ratio of GC content for codons influences the corresponding amino acid composition variation. Each genome has a preferred GC content, which promotes the variation of amino acid composition than the recruitment sequence of amino acids.

Old residuals have high levels of usage in all proteins, and new ones have rather low level of usage. However, the amino acids with lower levels of usage may cause the lower chance of amino acid replacement occurrence. Thus, more investigation is needed. The amino acid residuals were classified into three groups, the GC-rich group was given values of “2,” and the AT-rich group was given values of “0.” The group with average GC-content was given values of “1,” and these amino acids are D, V, S, E, L, T, Q, H, C, and W (Figure 3D). According to the amino acid recruitment order, these amino acids are further grouped into two sub-groups: group new, which include Q, H, C, W, and group old, which include D, V, S, E, L, T. The amino acid frequencies of “new” amino acids (Mean: 0.0191) are significantly lower than that of “old” ones (Mean: 0.0653, *P* < 2.2E–16). So these “old” amino acids do have higher frequencies than “lower” amino acids. However, the mean deviation of amino acids for “old” amino acid residuals is −0.0008, and that for “new” amino acids is 0.0048 (Figure 3E). The student’s *t*-test showed that the deviations of amino acids for “new” ones are significantly higher than that of the “old” ones (*P* < 2.2E–16) (Figure 3F). Thus, the amino acids with a lower levels of usage may not cause the lower chance of amino acid replacement occurrence. The conclusion we acquired here is reliable.

In conclusion, we determined LUCA genes/proteins, non-LUCA genes/proteins, and corresponding evolutionary rates with setting the MmarS2 as the LUCA. The principal component analysis showed that the first six components of compositions for 20 amino acids can be applied to build a significant linear models. The first principle component correlate well with GC contents of genes. Further analysis found that the GC contents correlate well with the *Ka* for genes, and the loss and gain of amino acids changes along with the GC contents. The amino acid frequencies coded by GC-rich codons positively correlate with the deviation of GC-content, while the compositions of amino acids coded by AT-rich codons negatively correlate with the deviation of the GC-content. Thus, the strong effect of GC contents on the protein evolution is proved. Next, we found that the recruitment order of amino acids has effects on amino acid composition during evolution. Its effect is weaker than that of GC content. Finally, as one feature of amino acids, GC contents has stronger effects on the protein evolution than other important features like recruitment order and cost.

## Discussion

Previously published research explained the gain and loss of amino acid variation during evolution with a neutral hypothesis, claiming that the trend in protein evolution was not driven by any simple trend at the DNA level (Jordan et al., 2005). In this paper, we investigated the evolution process of several orthologous genes and found that it was the GC content rather than amino acid recruitment order that determined the gain and loss of amino acid variation.

We used the conserved genes of MmarS2 as LUCA genes and investigated the evolution of both LUCA and non-LUCA genes. The MmarS2 is one of the possible LUCAs (Weiss et al., 2016). However, all lives are evolving after their first emergence. Thus, the MmarS2 genome we used may has a big difference from the origin. But the physiology and habitat for the LUCA is similar to that of MmarS2’s, and thus the evolution under such condition may not make it have a genome being biased too much. In addition, we chose those conserved essential genes for the investigation to make the results robust. According to the amino acid recruitment order, the amino acids Gln (Q), His (H), Cys (C), and Trp (W), which are encoded by codes of average GC content, should be newly recruited and thus should have lower compositions in LUCA. Fifty-one Methanoproducents (including MmarS2) have significantly lower Gln, His, and Trp contents than all other bacteria do (Figure 4). Meanwhile, *Clostridium*, the other possible LUCA phyla (Weiss et al., 2016) has similar amino acid compositions with Methanoproducents, and these four amino acids have higher contents in *Clostridium* proteomes than that of Methanoproducents. Thus, the MmarS2 could be considered as one closest species to LUCA. Next, we investigated the LUCA and non-LUCA genes, and concluded that the genomic composition and metabolic economy factors that shape relative amino acid compositions are qualitatively the same for LUCA and non-LUCA genes. The linear regression models also showed that both of them suffer strong selective power of GC contents during evolution. However, we chose essential LUCA proteins of MmarS2 for some analysis, which are supposed to be more conserved.

It was proved that GC contents contribute to the evolution rate as the main feature of amino acids. However, what cause the GC content variation? It was reported that GC contents are correlated with many genomic features like replication timing (Deschavanne and Filipski, 1995) and aerobiosis (Naya et al., 2002). The GC content of genes also depends on their positions in archaeal and bacterial genomes, and positions near the replication terminus tend to be A+T enriched in bacteria and G+C enriched in archaea (Daubin and Perrière, 2003). We do find that for those homologous genes to LUCA, there do exist a correlation between gene position and GC contents (significantly for 74% gene groups researched). The position also shows some correlation with the evolutionary rate *Ka* in this paper (only for 54% gene groups). The primary cause for the GC content variation may be the energy efficiency, which is the basic of evolution. For four amino acids encoded by GC-rich codons G, A, P, and R, the first two have lower costs and the last two have higher costs (the compositions of the later have lower compositions than the former from Figure 4). For amino acids encoded by AT-rich codons I, M, N, F, K and Y, N has a very low synthesis cost (Figure 3D and Supplementary Table S6). However, the average cost of GC rich amino acids is lower than that of AT-rich amino acids (Student’s *t*-test: *P* = 0.0306). Chen et al. (2016) reported that GC rich codons encode energetically cheaper amino acids, while at the same time G + C base pairs are more expensive than A +T/U pairs. A research in *Arabidopsis thaliana* and *Arabidopsis lyrata* showed that transcription-related mutations and GC content drive variation in nucleotide substitution rates (DeRose-Wilson and Gaut, 2007). The relationship between gene expression and GC-content in mammals was weak in some cases (Sémon et al., 2005). These researches may reflect that different trade-offs are related with energy efficiency. The GC content influences the evolutionary of proteins because of energy cost, and both the synthesis of bases and amino acids are involved in this process. Thus, highly GC-rich proteins may mean less cost for the synthesis of proteins, but also a higher cost for the synthesis of nucleotides. We observed a positive liner relationship between GC contents and *Ka* values for homologous genes from different genomes (Figure 1A). Corresponding *Ka* values grow with the GC contents of their homologous genes, and their homologous genes have GC contents close to genomes’ average. *Ka* is the ratio of the number of non-synonymous substitutions per non-synonymous site, thus higher *Ka* means more changes of amino acid sequences. It shows GC content correlates with the evolution process of amino acid sequences. Early proteins may have less choice of genetic codons for amino acids, and more AT rich codons means lower cost on transcriptions. Along with more and more codons for amino acids are recruited, the cost of amino acids is also under selection. The codons for amino acids have different GC content. Some amino acids like Ala, Arg, Gly, and Pro are GC rich, while Asn, Ile, Lys, Met, Phe, and Tyr are AT rich. During evolution, biased GC content will reflect on amino acid composition.

In this paper, we only surveyed the phenomenon among archaea and bacteria, and focused on few factors related to the evolution of amino acid composition including GC content, amino acid recruitment order, amino acid weight, and cost of amino acid synthesis. The factors influencing the amino acid composition in eukaryotes may be more complicate. Further investigation of the factors shaping amino acid composition during evolution is a basic problem to understand evolution. Additionally, more wet experiments would be involved in the future investigations for the evolution of amino acid composition.

## Materials and Methods

### Related Species and Genes

The LUCA gene list is obtained from the work of Weiss et al. (2016), which provides a relatively reliable LUCA gene set. The genome MmarS2 was downloaded from GenBank^1^ (Benson et al., 2003). Its essential gene list was acquired from DEG (Luo et al., 2014), a database of essential genes^2^. We determined the LUCA genes in the MmarS2 through the names of COGs or genes. There are 85 essential MmarS2 genes/proteins which are on the list of LUCA genes/proteins, which are seen as genes of a representative LUCA gene/protein list. Finally, we acquired sequences for these genes/proteins.

Next, we downloaded 2774 bacteria/archaea genomes from GenBank in 2017. Because Weiss et al. (2016) did not supply sequences for LUCA genes/proteins, we determined the LUCA genes in genomes through their gene names and/or COGs they belong to. There are 1196 genomes which have the same gene names as that of the LUCA gene/protein list, and there are only 124 genomes which have over 170 genes on the LUCA gene/protein list. Then, we acquired protein and ribonucleotide sequences of genes having same gene names to LUCA genes in these genomes. After that, we need to pair them with genes from the representative LUCA gene list we determined before. Here, we used the sequence alignment tool BLAST to pair these genes (Altschul et al., 1990), and we found out homologous proteins of MmarS2 in 527 genomes (1196 genomes minus 669 genomes whose proteins have same names as that of LUCA proteins but are not homologous to those from representative LUCA protein list) (Supplementary Table S3).The similar procedure were used to determine homologous proteins of non-LUCA proteins in MmarS2.

Finally, we got homologous proteins of 90 non-LUCA and 23 LUCA, from which we can observe the variations of amino acid compositions and evolutionary rate *Kas* among proteins. These results were used further to compare the evolutionary result of amino acid compositions for proteins among genomes and among proteins.

### Evolutionary Rates

Orthologous gene pairs were identified based on reciprocal best hits using the protein-protein basic local alignment search tool BLAST service Blastp^3^ (Altschul et al., 1990; McGinnis and Madden, 2004) with criteria of *E* < 10^−5^, 80% minimum residues that could be aligned, and at least 30% identity. A local blast database was constructed with sequences of researched proteome. Every two proteomes (One is of MmarS2, which only includes those essential LUCA proteins) were used to find homologous genes in each turn, and during the BLAST step, the two proteomes were used to construct local BLAST database in turn. The acquired lists of homologous proteins are highly reliable. Protein sequences encoded by identified orthologous gene pairs were aligned with ClustalW (Larkin et al., 2007), and then back-translated into nucleotide sequences based on their original sequences. Numbers of substitutions per non-synonymous site (*Ka*) and numbers of substitutions per synonymous site (*Ks*) were calculated using the PAML package (Yang, 1997, 2007) with default parameters (yn00 model was employed).

### Deviation of Amino Acids and Deviation of GC Contents

We used the deviation of amino acid to depict the gain and loss of amino acids. If the deviation of amino acid is negative, it means the ratio of special amino acid is decreased/lost. If the deviation of amino acid is positive, it means the ratio of special amino acid is increased/gained. Amino acid composition for each protein was calculated. There are 20 standard amino acids. The ratio of each amino acid in the protein sequence is equal to the amino acid frequency. The deviation of amino acids is the difference between the amino acid composition of one protein to its homologous protein. We use the amino acid composition of proteins in the 65 genomes to minus the amino acid composition of proteins in MmarS2 to acquire the deviation of amino acids. This value reflects the gain and loss ratios for the twenty standard amino acids, and also reflects the evolution selection on amino acid compositions.

Each gene for these proteins has a different GC content. We use the GC contents of genes in the 65 genomes to minus the GC contents of genes in MmarS2 to acquire the deviation of GC contents. This value reflects the tendency of GC content for genes in different genomes, which could be under selection.

### Amino Acid Features

The analyses are based on the GC feature of codons for special amino acid, the amino acid recruitment order, the molecular weight, and the energy cost for synthesizing special amino acid.

Some amino acids are encoded by GC-rich codons, and some are by AT-rich codons. Thus, if the first two bases of the codons are mainly G and C, the amino acid will be grouped to GC-rich group, and if the first bases two of the codons are mainly A and T, the amino acid will be grouped to AT-rich group. The rest amino acids are grouped into another groups. We give GC-rich amino acids value of 2, AT-rich amino acids value of 1 and others 0. The GC content for amino acid A, C, E, D, G, F, I, H, K, M, L, N, Q, P, S, R, T, W, V, and Y are 2, 1, 1, 1, 2, 0, 0, 1, 0, 0, 1, 0, 1, 2, 1, 2, 1, 1, 1, and 0.

The amino acid recruitment order is the order of amino acids originated during the long term evolution, which was acquired from the work of Jordan et al. (2005). To exclude the effect of GC content, we compared the special “old” (D, S, E, L) and “new” (Q, H, C, W) amino acid residues, which are encoded by codons with average GC content.

The amino acid cost, which is energy cost for synthesizing amino acids, are different. We used the amino acid cost from *E. coli* (Akashi and Gojobori, 2002). All feature values are provided in Supplementary Table S6.

### Software

We used basic local alignment search tool BLAST to find homologs or for similarity search (Altschul et al., 1990; McGinnis and Madden, 2004), ClustalW to align sequences (Thompson et al., 1994), and PAML to calculate evolutionary rate *Ka*. All these software /packages were called through Python scripts (Oliphant, 2007). We also used R version 2.7 to do statistical analysis^4^ such as the principal component analysis and correlation analysis. We extracted the six principal components and then used the least square method to construct a linear model between them and the evolutionary rate *Ka*. All correlation analyses are based on Pearson correlation.

## Author Contributions

FBG conceived and coordinated the project. MZD designed and implemented the computational analyses and WW double-checked these analyses. CZ, HW, and SL downloaded and analyzed the data. FBG, WW, and MZD discussed the results. MZD wrote the paper, assisted by the other authors. All the authors read and approved the final manuscript.

## Conflict of Interest Statement

The authors declare that the research was conducted in the absence of any commercial or financial relationships that could be construed as a potential conflict of interest.

## Footnotes

1 ftp://ftp.ncbi.nlm.nih.gov/genomes/genbank/archaea/Methanococcus_maripaludis/

2 http://www.essentialgene.org

3 https://blast.ncbi.nlm.nih.gov/Blast.cgi

4 https://www.r-project.org/

## Supplementary Material

TABLE S1 The organism name and corresponding number of homologous COGs to LUCA.

TABLE S2 Functional and taxonomic characterization of the LUCA’s gene families potentially present in LUCA.

TABLE S3 Lists of organisms and corresponding numbers of orthologous proteins to MmarS2.

TABLE S4 Principal component regression between evolutionary rates and amino acid compositions for MmarS2 proteins.

TABLE S5 The deviation of GC content and amino acids for protein coding genes homologous to LUCA genes.

TABLE S6 Types of feature values for amino acids.

FIGURE S1 The genome sizes positively correlate with the GC content in genes.

FIGURE S2 Ancient amino acids have higher ratio than newly recruited ones in proteomes.
