# Supplementary material for: The GC Content as a Main Factor Shaping the Amino Acid Usage During Bacterial Evolution Process
Source: Front Microbiol. 2018 Dec 7;9:2948. doi: 10.3389/fmicb.2018.02948 (PMC6292993; doi:10.3389/fmicb.2018.02948)
Supplement: TABLE S6 — Types of feature values for amino acids. [file Data_Sheet_6.docx]

**Supplementary Table S6**

|  | **GC** | **Recruiment order*** | **Energy cost*** | **Molecular weight*** |
| --- | --- | --- | --- | --- |
| **A** | 2 | 2 | 11.7 | 89 |
| **C** | 1 | 16 | 24.7 | 121 |
| **E** | 1 | 7 | 15.3 | 147 |
| **D** | 1 | 3 | 12.7 | 133 |
| **G** | 2 | 1 | 11.7 | 75 |
| **F** | 0 | 17 | 52 | 165 |
| **I** | 0 | 12 | 32.3 | 131 |
| **H** | 1 | 14 | 38.3 | 155 |
| **K** | 0 | 15 | 30.3 | 146 |
| **M** | 0 | 19 | 34.3 | 149 |
| **L** | 1 | 8 | 27.3 | 131 |
| **N** | 0 | 13 | 14.7 | 132 |
| **Q** | 1 | 11 | 16.3 | 146 |
| **P** | 2 | 5 | 20.3 | 115 |
| **S** | 1 | 6 | 11.7 | 105 |
| **R** | 2 | 10 | 27.3 | 174 |
| **T** | 1 | 9 | 18.7 | 119 |
| **W** | 1 | 20 | 74.3 | 204 |
| **V** | 1 | 4 | 23.3 | 117 |
| **Y** | 0 | 18 | 50 | 181 |

*These values are from other researchers’ published work. The references

could be found in the paper
