# Supplementary figures and images for: The GC Content as a Main Factor Shaping the Amino Acid Usage During Bacterial Evolution Process
Source: Front Microbiol. 2018 Dec 7;9:2948. doi: 10.3389/fmicb.2018.02948 (PMC6292993; doi:10.3389/fmicb.2018.02948)

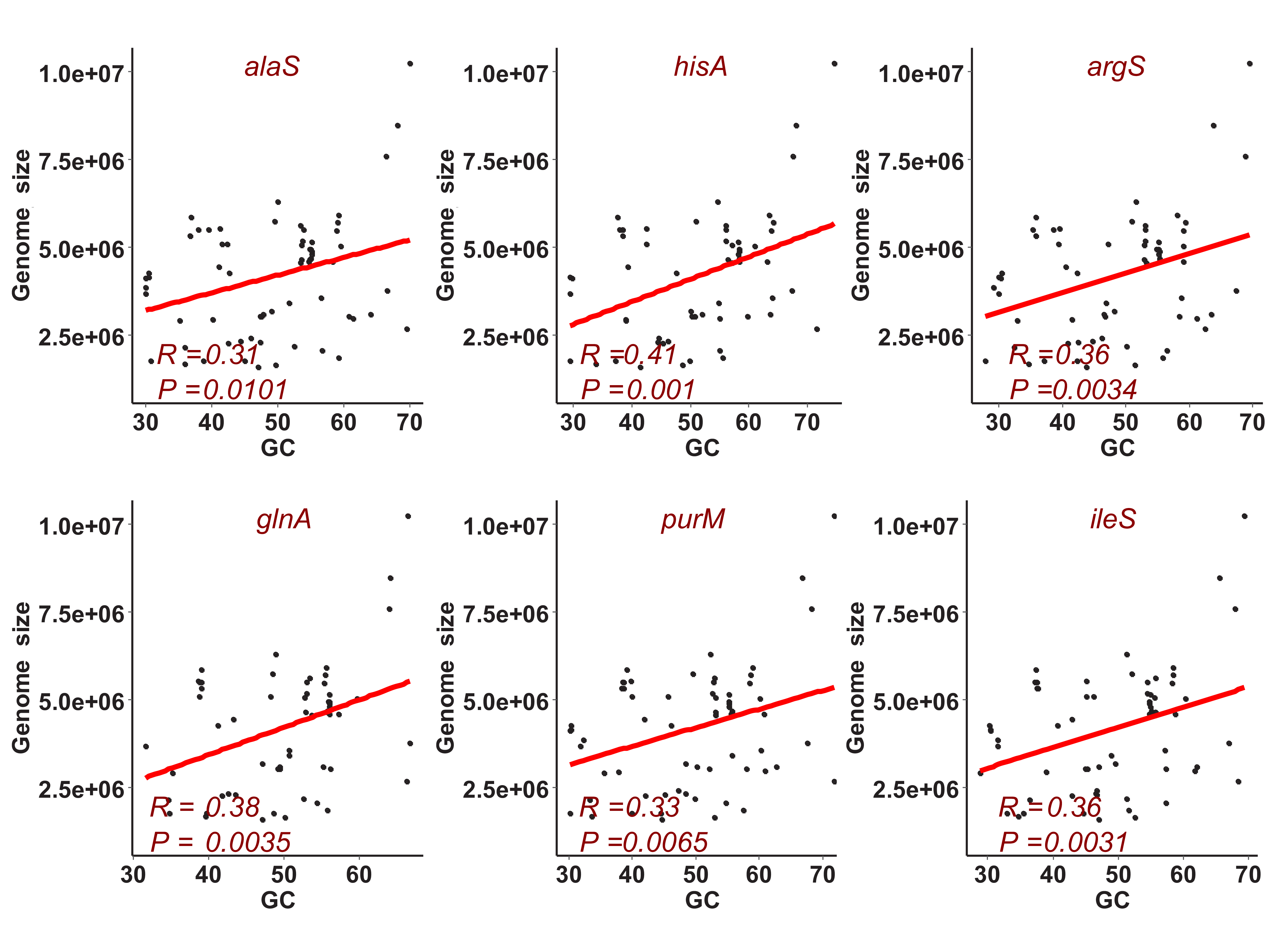

Supplement: FIGURE S1 — The genome sizes positively correlate with the GC content in genes. [file Image_1.JPEG]

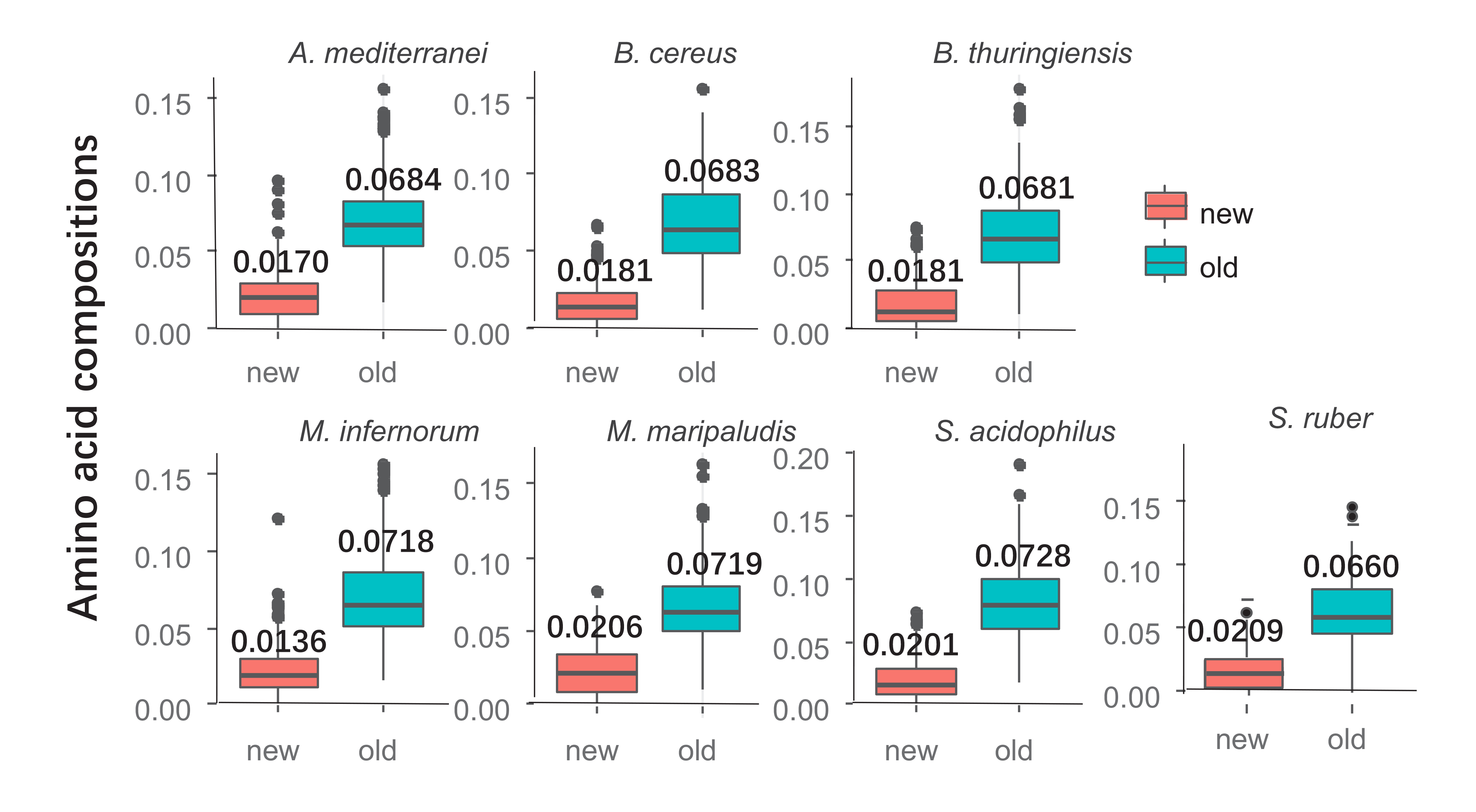

Supplement: FIGURE S2 — Ancient amino acids have higher ratio than newly recruited ones in proteomes. [file Image_2.JPEG]
